# Bradykinin regulates cell growth and migration in cultured human cardiac c-Kit+ progenitor cells

**DOI:** 10.18632/oncotarget.14609

**Published:** 2017-01-12

**Authors:** Gang Li, Yan Wang, Gui-Rong Li

**Affiliations:** ^1^ Xiamen Cardiovascular Hospital, Xiamen University, Xiamen, Fujian, China; ^2^ Department of Medicine, Li Ka Shing Faculty of Medicine, University of Hong Kong, Pokfulam, Hong Kong, China

**Keywords:** Bradykinin, bradykinin receptor 2, human cardiac c-Kit+, progenitor cells, proliferation, migration, Pathology Section

## Abstract

Bradykinin is a well-known endogenous vasoactive peptide. The present study investigated the bradykinin receptor expression in human cardiac c-Kit^+^ progenitor cells and the potential role of bradykinin in regulating cell cycling progression and mobility. It was found that mRNA and protein of bradykinin type 2 receptors, but not bradykinin type 1 receptors, were abundant in cultured human cardiac c-Kit^+^ progenitor cells. Bradykinin (1-10 nM) stimulated cell growth and migration in a concentration-dependent manner. The increase of cell proliferation was related to promoting G0/G1 transition into G2/M and S phase. Western blots revealed that bradykinin significantly increased pAkt and pERK1/2 as well as cyclin D1, which were countered by HOE140 (an antagonist of bradykinin type 2 receptors) or by silencing bradykinin type 2 receptors. The increase of pAkt, pERK1/2 and cyclin D1 by bradykinin was prevented by the PI3K inhibitor Ly294002, the PLC inhibitors U73122 and neomycin, and/or the PKC inhibitor chelerythrine and the MAPK inhibitor PD98059. Our results demonstrate the novel information that bradykinin promotes cell cycling progression and migration in human cardiac c-Kit^+^ progenitor cells via activating PI3K, PLC, PKC, cyclin D1, pERK1/2, and pAkt.

## INTRODUCTION

Cardiac c-Kit^+^ progenitor cells can potentially differentiate into at least three main cardiac cells, i.e. myocytes, vascular smooth muscle cells and endothelial cells [1], and are therefore believed to be a viable cell source of cell-based therapeutic strategy for treating myocardial ischemia/reperfusion injury in animal models [2–4] and patients with ischemic cardiomyopathy [5–8]. It is generally recognized that stem cells from implanted or residential source must migrate to the injured site before they can proliferate then differentiate into cardiac myocytes and/or endothelial cells to repair the damaged myocardial tissue [9]. Therefore, proliferation and migration of cardiac c-Kit^+^ progenitor cells are essential in myocardial repair. However, regulation of cell cycling progression and migration is not fully understood in human cardiac c-Kit^+^ progenitor cells.

Bradykinin is a principal active agent of tissue kinin-kallikrein system and exerts its major effects through the activation of the two types of bradykinin receptors, B1Rs and B2Rs [10]. Previous studies have demonstrated that bradykinin increases cell proliferation in cultured bovine corneal endothelial cells [11], canine corneal epithelial cells [12], rabbit corneal fibroblasts [13], rat aortic vascular smooth cells, and amoeboid migration of cancer cells [14]. Bradykinin has been reported to be cardioprotective [15–17]; however, it is unknown whether cell cycling progression and migration of cardiac c-Kit^+^ progenitor cells are involved in bradykinin-induced cardioprotection. The present study was therefore designed to determine the expression of bradykinin receptors in cultured human cardiac c-Kit^+^ progenitor cells and to investigate whether/how cell growth and mobility are regulated by bradykinin. Our results showed that B2Rs, but not B1Rs, were present in human cardiac c-Kit^+^ progenitor cells. Bradykinin enhanced cell cycling progression and migration by increasing cyclin D1, pAkt and pERK1/2 *via* activating B2Rs.

## RESULTS

### Expression of bradykinin receptors in human cardiac c-Kit^+^ progenitor cells

**Figure 1:**
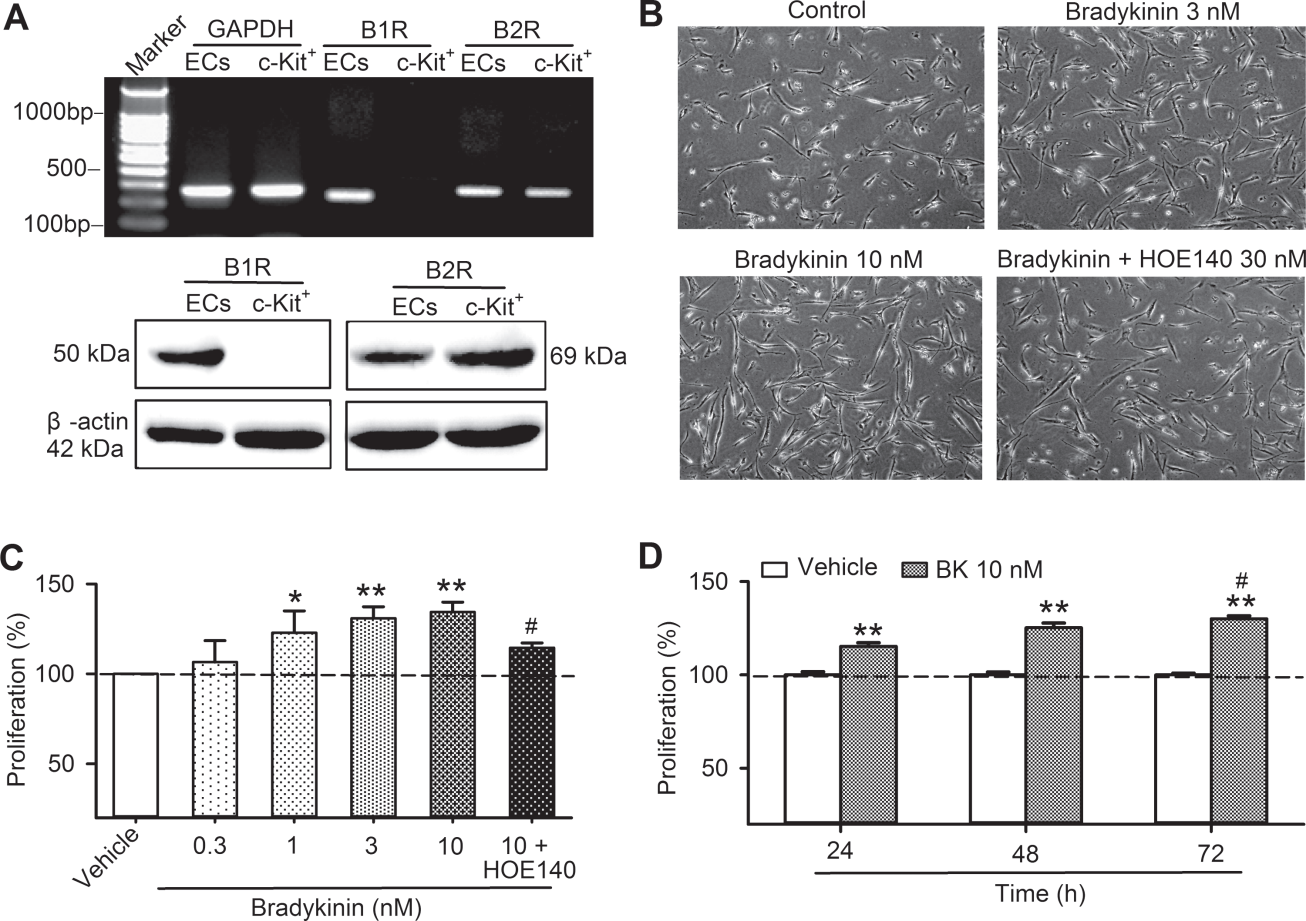
The expression of bradykinin receptors and bradykinin effect on cell proliferation in human cardiac c-Kit^+^ progenitor cells **A**. RT-PCR image (upper panel) and western blots (lower panel) of B1Rs and B2Rs in human cardiac c-kit^+^ progenitor cells and in human umbilical vein endothelial cells (ECs) treated with IL-1β. **B**. Images of cells culture with vehicle (control), bradykinin (3 or 10 nM) or 30 nM HOE140 plus 10 nM bradykinin (BK). **C**. Percentage values of cell proliferation determined with MTT assay in human cardiac c-kit^+^ progenitor cells treated with vehicle (control), bradykinin (0.3-10 nM) or 30 nM HOE140 plus 10 nM bradykinin (48 h, *n* = 6, *P < 0.05,** *P <* 0.01, *vs* control group; #*P <* 0.05, *vs* 10 nM bradykinin). **D**.. Percentage values of cell proliferation in human cardiac c-Kit^+^ progenitor cells treated with vehicle (control) or bradykinin (10 nM) for 24, 48, and 72 h (*n* = 4, **P < 0.01 *vs*. vehicle, #*P* < 0.05 *vs*. 24 h).

Messenger RNA (mRNA) and protein of B1Rs and B2Rs were determined in human cardiac c-Kit^+^ progenitor cells using RT-PCR and Western blotting analysis, respectively. Figure 1A shows that mRNA expression of B2Rs, but not B1Rs, was present in human cardiac c-Kit^+^ progenitor cells. Protein expression of B2Rs was confirmed with Western blot analysis. As controls, mRNAs and proteins of both B1Rs and B2Rs were expressed in human umbilical vein endothelial cells treated with interleukin 1β (IL-1β) 1 ng/ml for 24 h. These results suggest that B2Rs, but not B1Rs is expressed in human cardiac c-Kit^+^ progenitor cells.

### Effects of bradykinin on cell cycling progression in human cardiac c-kit^+^ progenitor cells

Figure 1B shows images of cultured human cardiac c-Kit^+^ progenitor cells in the absence and presence of bradykinin or bradykinin plus the B2R antagonist HOE140. The cell density was clearly increased with bradykinin (3 and 10 nM) incubation (48 h), but not with bradykinin plus HOE140 (30 nM), suggesting that bradykinin may stimulate cell proliferation in human cardiac c-Kit^+^ progenitor cells.

The effects of bradykinin on cell proliferation and cell cycling progression were further analyzed. Figure 1C shows the percentage of cell proliferation determined with MTT method in the presence of different concentrations of bradykinin. Bradykinin at 0.3 -10 nM (48 h incubation) increased cell proliferation in a concentration-dependent manner. Significant proliferation enhancement was observed at 1, 3 and 10 nM (*n* = 6, *P* < 0.05 or *P* < 0.01 *vs*. control), and the effect was reversed by the B2R antagonist HOE140 (*P* < 0.05 *vs*. 10 nM bradykinin), and the effect was time-dependent (Figure 1D).

**Figure 2:**
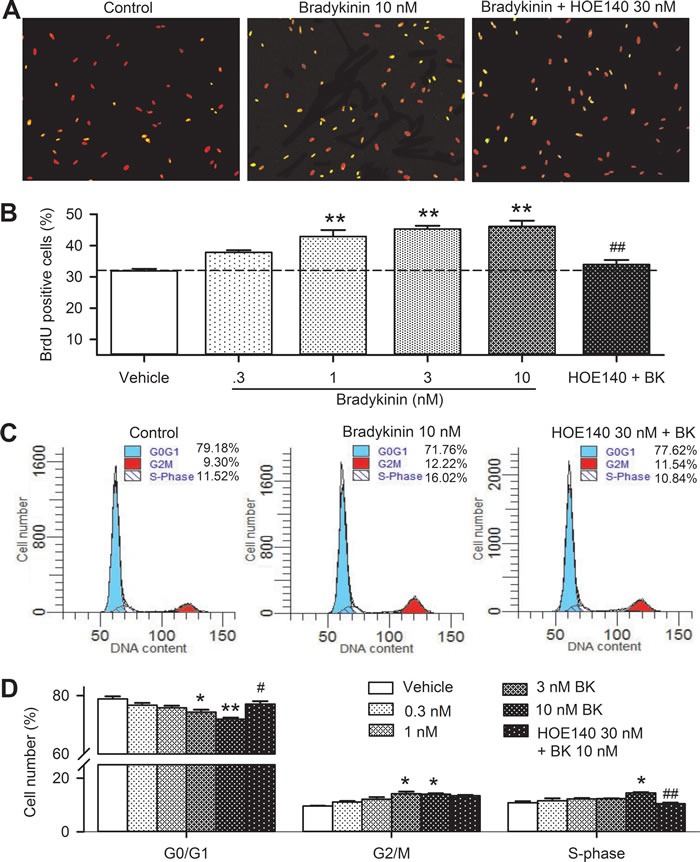
Effects of bradykinin (BK) on cell cycling progression in human cardiac c-Kit^+^ progenitor cells **A**. Immunofluorescent staining of BrdU with anti-BrdU antibody in human cardiac c-Kit^+^ progenitor cells treated with vehicle (control), 10 nM bradykinin or bradykinin plus 30 nM HOE140 for 48 h. The nuclei are labelled by PI staining in red, the proliferative cells are labelled by BrdU staining (merged with PI in yellow). **B**. Percentage values of BrdU positive nuclei in cells treated with vehicle (control) or different concentrations of bradykinin (0.3-10 nM) or 30 nM HOE140 plus 10 nM bradykinin. **C**. Flow cytometry graphs showing distribution of cell cycle phases treated with vehicle, 10 nM bradykinin or bradykinin plus 30 nM HOE140. **D**. Percentage values of cell cycling population at different phases in cells treated with corresponding bradykinin (*n* = 6, *P < 0.05, **P < 0.01 *vs* control group; #*P <* 0.05, ##*P <* 0.01 *vs* 10 nM bradykinin).

The effect of bradykinin on proliferation of human cardiac c-Kit^+^ progenitor cells was confirmed by BrdU incorporation assay (Figure 2A). BrdU positive cells were increased with bradykinin incubation. Figure 2B shows that cell number with BrdU positive nuclei was increased by bradykinin at 0.3-10 nM. The cells with BrdU positive nuclei were 29.8% in control, and increased to 40.6% with 10 nM bradykinin (*n* = 6, *P* < 0.01), and the effect was countered by co-application of the B2Rs antagonist HOE140 (30 nM, *n* = 6, *P* < 0.01 *vs*. bradykinin).

The effect of bradykinin on cell cycle progression was determined with flow cytometry in human c-Kit^+^ progenitor cells. Figure 2C shows the representative flow cytometry graphs with cell cycle distribution in cells treated with vehicle (control), 10 nM bradykinin, and bradykinin plus 30 nM HOE140 for 48 h. The number of cells in G0/G1 phase was reduced, while the number of cells in G2/M and S-phase were increased by bradykinin and the effects were reversed in cells treated with both bradykinin and HOE140. Figure 2D illustrates the percentage of cycling progression phases in cells treated with vehicle or 0.3, 1, 3, and 10 nM bradykinin or 10 nM bradykinin plus 30 nM HOE140. Bradykinin reduced the proportion of cells at G0/G1 boundary in a concentration-dependent manner. The proportion of G0/G1 boundary was decreased from 79.6±0.6% in control to 71.5±1.5% in cells treated with 10 nM bradykinin (*n* = 6, *P <* 0.01 *vs*. control), while the cell proportion of S-phase was increased from 10.8±0.6% in control to 14.5±1.3 % (*P* < 0.05 *vs*. control). These effects were reversed by the B2R antagonist HOE140 (*P <* 0.01 *vs*. 10 nM bradykinin). These results suggest that bradykinin enhances the proliferation of human cardiac c-Kit^+^ progenitor cells *via* activating B2Rs by promoting the G0/G1 boundary to S phase.

### Effect of bradykinin on migration of human cardiac c-Kit^+^ progenitor cells

**Figure 3:**
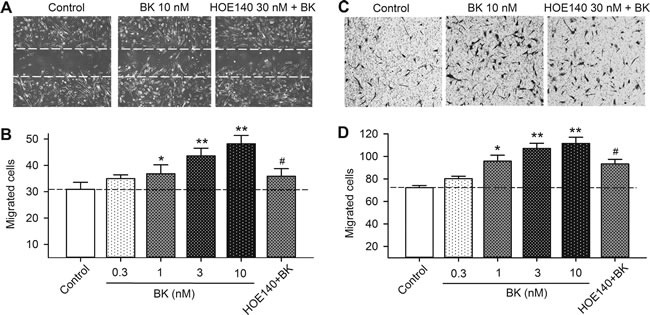
Effect of bradykinin (BK) on cell mobility in human cardiac c-Kit^+^ progenitor cells **A**. Representative images of wound healing assay in cells treated with vehicle (control), 10 nM bradykinin, or bradykinin plus 30 nM HOE140 for 8 h. **B**. Migrated cells into acellular area in cells treated with vehicle, different concentrations of bradykinin (0.3-10 nM) or 10 nM bradykinin plus 30 nM HOE140. **C**. Images of migrated cells on the lower surface membrane in Transwell assay in human cardiac c-Kit^+^ progenitor cells treated with vehicle, 10 nM bradykinin, or bradykinin plus 30 nM HOE140. **D**. Migrated human cardiac c-Kit^+^ progenitor cells on the lower surface membrane when treated with vehicle, bradykinin (0.3-10 nM), or 10 nM bradykinin plus 30 nM HOE140. (*n* = 6, *P < 0.05, **P < 0.01, *vs* control group; #*P <* 0.05, *vs* 10 nM bradykinin).

To determine whether bradykinin increases cell migration in human cardiac c-Kit^+^ progenitor cells, wound healing and chemotaxis assays were conducted in cells treated with different concentrations of bradykinin. Figure 3A shows the wound healing images in cells treated with vehicle, 10 nM bradykinin or the B2R antagonist HOE140 (30 nM) plus 10 nM bradykinin. Bradykinin increased the cell migration, and the effect was countered by HOE140. Figure 3B illustrates the migrated cells into the acellular area in cells treated with 0.3, 1, 3 and 10 nM bradykinin or 30 nM HOE140 plus 10 nM bradykinin. Bradykinin increased cell migration in a concentration-dependent manner (*n* = 6, *P <* 0.05 or *P <* 0.01 *vs*. control), and the effect was countered by HOE140 (*n* = 6, *P <* 0.05 *vs*. 10 nM bradykinin).

The effect of bradykinin on migration was further determined with transwell assay to limit the potential contamination by cell proliferation. Figure 3C shows the images of migrated cells on the lower surface membrane in cells treated with vehicle, 10 nM bradykinin, and bradykinin plus 30 nM HOE140. The migrated cell number was increased in cells treated with bradykinin, and the effect was reduced by HOE140. The number of migrated cells is illustrated in Figure 3D. The cell migration, as in wound-healing assay, was increased by bradykinin in a concentration-dependent manner (*n* = 6, *P <* 0.05 or *P* < 0.01 *vs*. control), and the effect was countered by 30 nM HOE140 (*P <* 0.01 *vs*. 10 nM bradykinin). These results indicate that bradykinin increases the mobility of human cardiac c-Kit^+^ progenitor cells *via* activating B2Rs.

### Bradykinin effects on cell proliferation, cycling progression and mobility after knock-down of B2Rs

**Figure 4:**
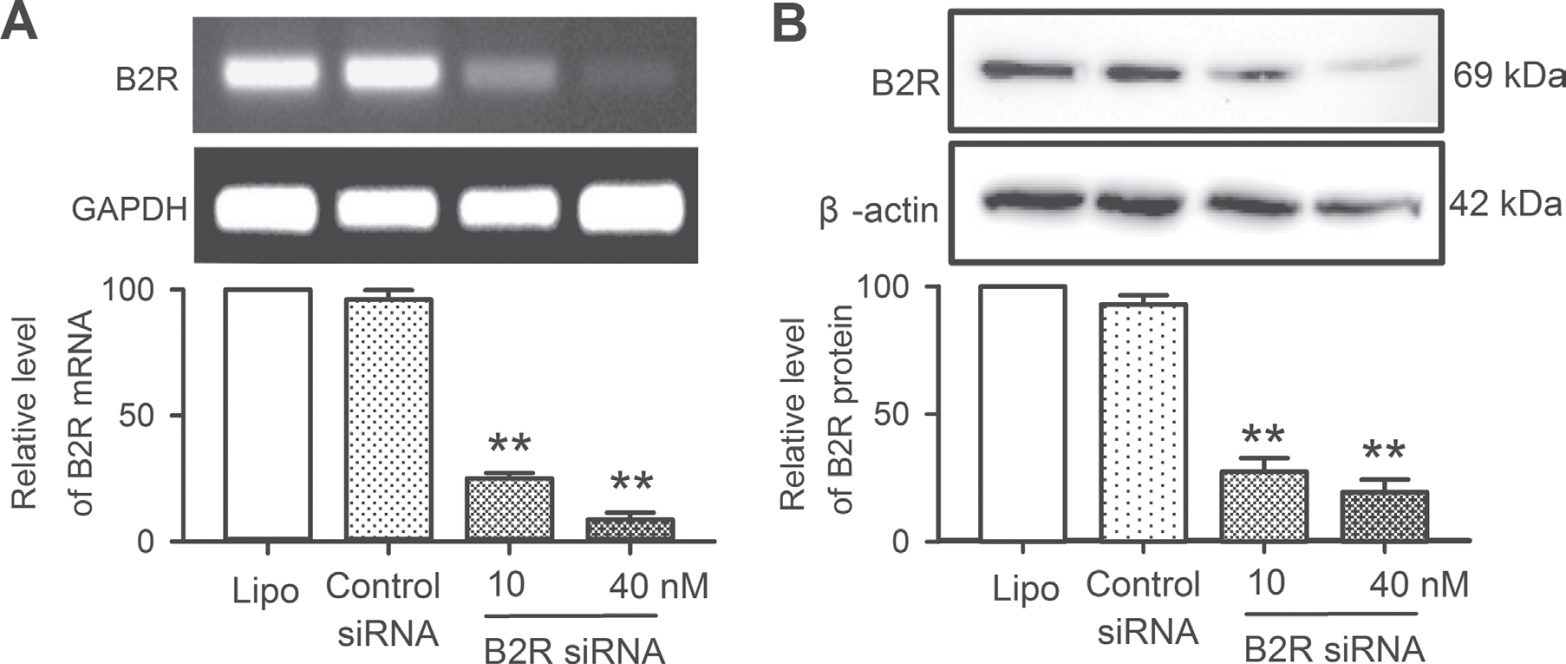
Messenger RNA and protein of B2Rs in human cardiac c-Kit^+^ progenitor cells transfected with B2R siRNA **A**.. B2Rs mRNA level determined with RT-PCR in cells transfected with Lipofectamine 2000 (Lipo), control siRNAs or B2R siRNA (10 and 40 nM) for 48 h. **B**.. Western blots of B2Rs in human cardiac c-Kit^+^ progenitor cells transfected with Lipofectamine 2000, control siRNAs or B2R siRNA (10 and 40 nM) for 72 h. *n* = 3, **P < 0.01 *vs*. control siRNA.

The effects of bradykinin on cell proliferation, cell cycling progression and mobility were determined in human cardiac c-Kit^+^ progenitor cells with B2Rs silenced. Figure 4 illustrates the silencing efficiency in human cardiac c-Kit^+^ progenitor cells transfected with B2R siRNA. The mRNA (Figure 4A) and protein levels (Figure 4B) of B2Rs were remarkably reduced in cells transfected with B2R siRNA (10 or 40 nM) for 48 h or 72 h (*n* = 3, *P <* 0.01 *vs*. 40 nM control siRNA).

**Figure 5:**
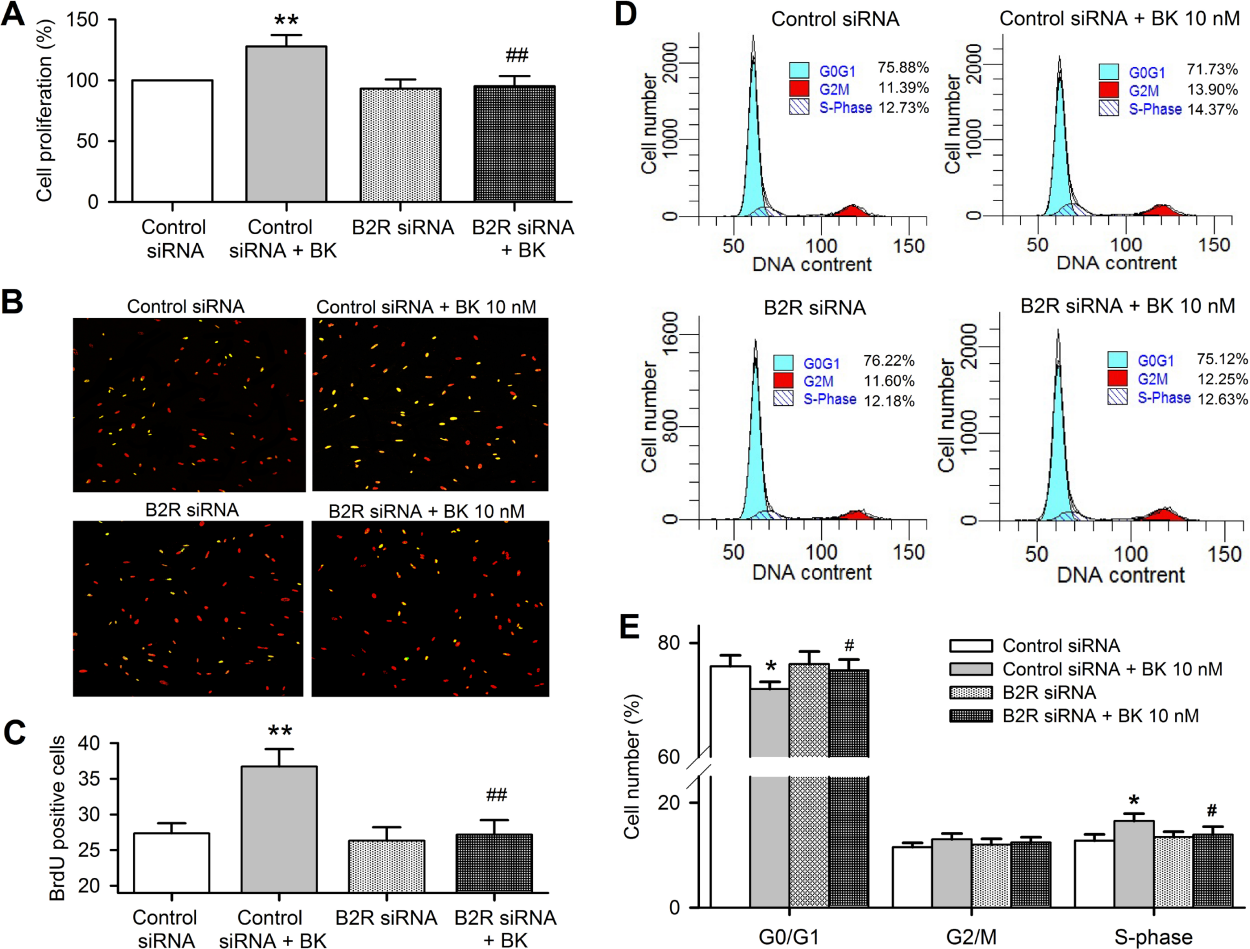
Bradykinin (BK) effects on cell cycling progression in human cardiac c-Kit^+^ progenitor cells with B2Rs silenced **A**. Cell proliferation determined with MTT in cells transfected with control siRNA and B2R siRNA (40 nM, *n* = 6). **B**. Images of BrdU incorporation in cells transfected with control siRNA or B2R siRNA with or without 10 nM bradykinin treatment. The nuclei are labelled by PI staining in red, the proliferative cells are labelled by BrDu staining (merged with PI in yellow). **C**. Percentage values of BrdU incorporation. **D**. Representative flow cytometry graphs in cells transfected with control siRNA or B2R siRNA with or without 10 nM bradykinin treatment. **E**. Percentage values of cell population at different cycling phases. (*n* = 6, *P < 0.05, **P < 0.01 *vs* control siRNA, #*P <* 0.05, ##*P <* 0.01 *vs*. control siRNA plus 10 nM bradykinin).

MTT and BrdU incorporation assays were used for determining the effect of bradykinin on cell proliferation in human cardiac c-Kit^+^ progenitor cells transfected with B2R siRNA. Figure 5A shows that cell proliferation was increased by bradykinin in cells transfected with control siRNA (*n* = 6, *P <* 0.01), but not in cells transfected with B2R siRNA (*P <* 0.01 *vs*. control siRNA). Similar results were observed in BrdU assay (Figure 5B and 5C, P *<* 0.01).

The cell cycling progression was determined by flow cytometry analysis after silencing of B2Rs and bradykinin treatment (Figure 5D). Bradykinin decreased G0/G1 population and increased S-phase population in cells transfected with control siRNA (Figure 5E, *n* = 6, *P <* 0.05), but not in cells transfected with B2R siRNA. These results indicate that bradykinin promotes cell cycling progression *via* activating B2Rs in human cardiac c-Kit^+^ progenitor cells.

**Figure 6:**
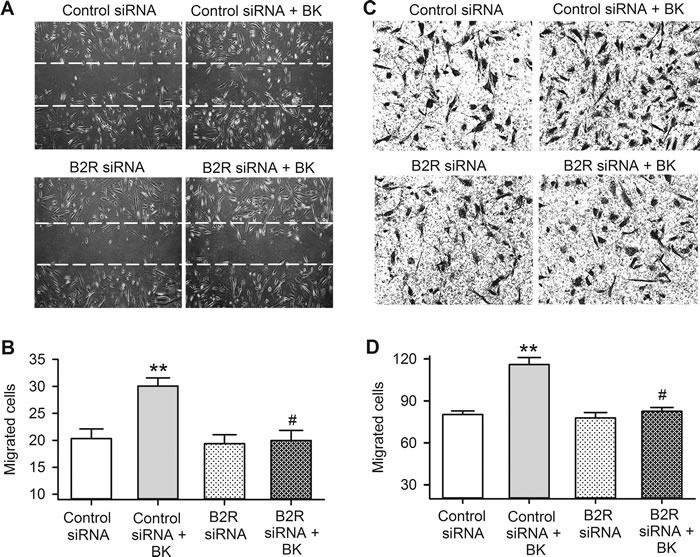
Bradykinin (BK) effect on migration in human cardiac c-Kit^+^ progenitor cells with B2Rs silenced **A**. Representative images of wound healing assay in cells transfected with controls siRNA or B2R siRNA with or without 10 nM bradykinin treatment for 8 h. **B**. Cell number migrated into the acellular area in cells transfected corresponding siRNAs and with 10 nM bradykinin treatment. **C**. Images of migrated cells on the lower surface membrane in transwell assay in human cardiac c-Kit^+^ progenitor cells transfected with control siRNA or B2R siRNA molecules, with or without 10 nM bradykinin treatment. **D**. Migrated cell number on the lower surface membrane. (*n* = 6, ** *P <* 0.01 *vs* control siRNA, # *P <* 0.05 *vs*. control siRNA plus 10 nM bradykinin)

The effects of bradykinin on cell migration were determined with wound healing assay (Figure 6A and 6B) and transwell assay (Figure 6C and 6D) in cells transfected with 40 nM B2R siRNA molecules. Cells migration to the acellular area or the lower membrane surface of transwell was increased by bradykinin in human cardiac c-Kit^+^ progenitor cells transfected with control siRNA (*P <* 0.01), but not in cells transfected with B2R siRNA. These results indicate that cell migration induced by bradykinin is mediated by B2Rs in human cardiac c-Kit^+^ progenitor cells.

### Signal molecules involved in bradykinin regulation of cell cycling progression and migration

**Figure 7:**
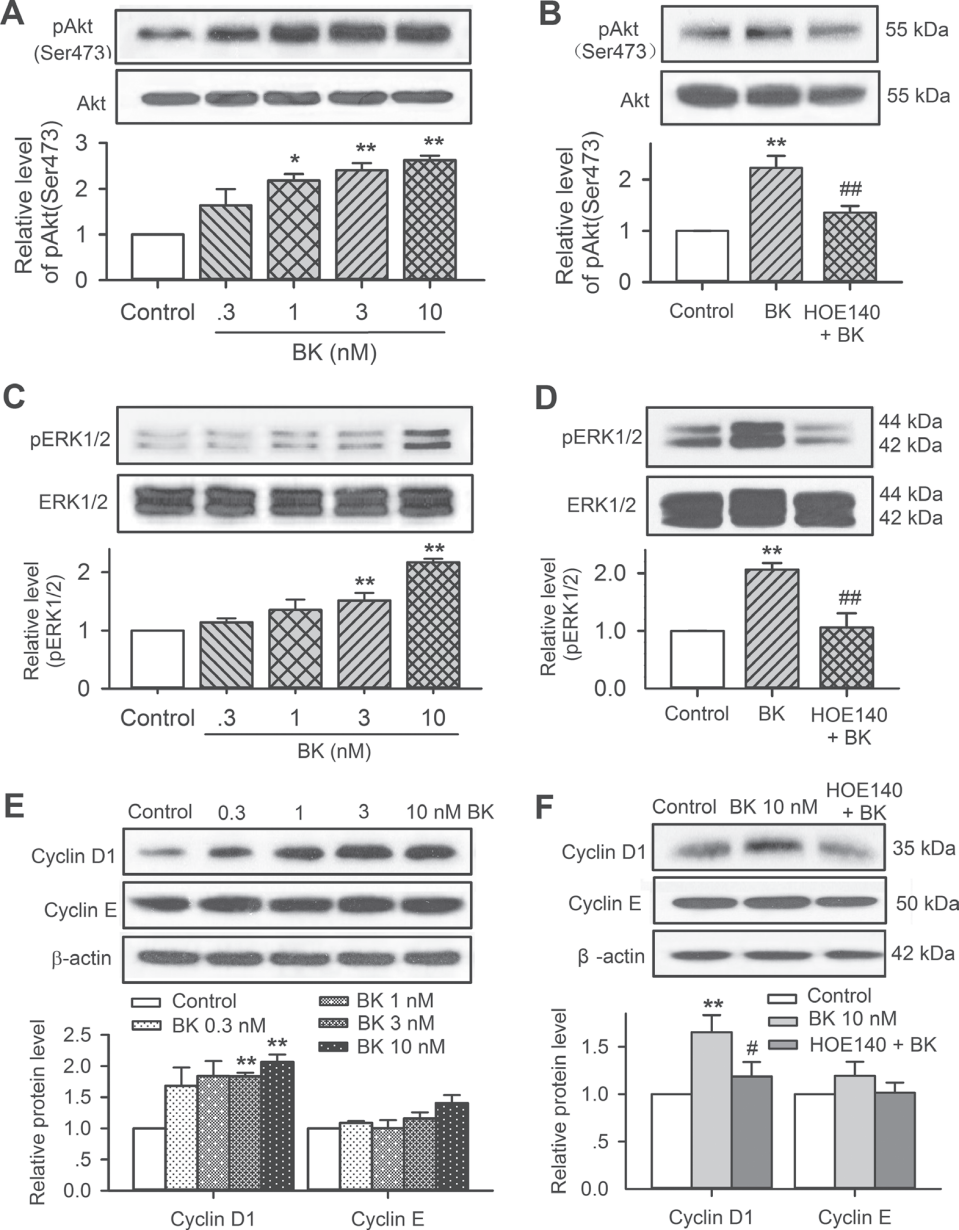
Involvement of molecular signals in regulating proliferation and migration in c-Kit^+^ progenitor cells **A**. Western blots of pAkt(Ser473) and Akt in cells treated with 0.3-10 nM bradykinin (*n* = 3,* *P <* 0.05, **P < 0.01 *vs*. vehicle control). **B**. HOE140 (30 nM) reduced the increased pAkt by 10 nM bradykinin (*n* = 3, **P < 0.01 *vs*. vehicle control; ##*P <* 0.01 *vs*. 10 nM bradykinin). **C**. Increase of pERK1/2 level in cells treated with 0.3-10 nM bradykinin (*n* = 3, **P < 0.01 *vs*. vehicle control). **D**. HOE140 (30 nM) reversed the increase of pERK1/2 by 10 nM bradykinin (*n* = 3, **P < 0.01 *vs*. vehicle control; ##*P <* 0.01 *vs*. 10 nM bradykinin). **E**. Increase of cyclin D1 level (but not cyclin E) in cells treated with 0.3-10 nM bradykinin (*n* = 3, **P < 0.01 *vs*. vehicle control). **F**. HOE140 (30 nM) reversed the increase of cyclin D1 by 10 nM bradykinin (*n* = 3, **P < 0.01 *vs*. vehicle control; ##*P <* 0.01 *vs*. 10 nM bradykinin).

To determine the signal molecules involved in bradykinin regulation of cell cycling progression and migration, the molecules related to cell mobility and cell cycling progression were determined in human cardiac c-Kit^+^ progenitor cells. Figure 7 illustrates the Western blot analysis for the survival kinase pAkt, the mitogen-activated protein kinase pERK1/2, and the cell cycling-related proteins cyclin D1 and cyclin E in human cardiac c-Kit^+^ progenitor cells treated with different concentrations of bradykinin. It is interesting to note that pAkt (Ser473), pERK1/2, and cyclin D1, but not cyclin E were increased by bradykinin in a concentration-dependent manner. The increase of these kinases by bradykinin was fully reversed in cells treated with the B2R antagonist HOE140 (*n* = 3, *P <* 0.01 *vs*. bradykinin alone). These results suggest that activation of pAkt, pERK1/2 and cyclin D1 are involved in regulation of cell proliferation and/or migration by bradykinin *via* activating B2Rs.

**Figure 8:**
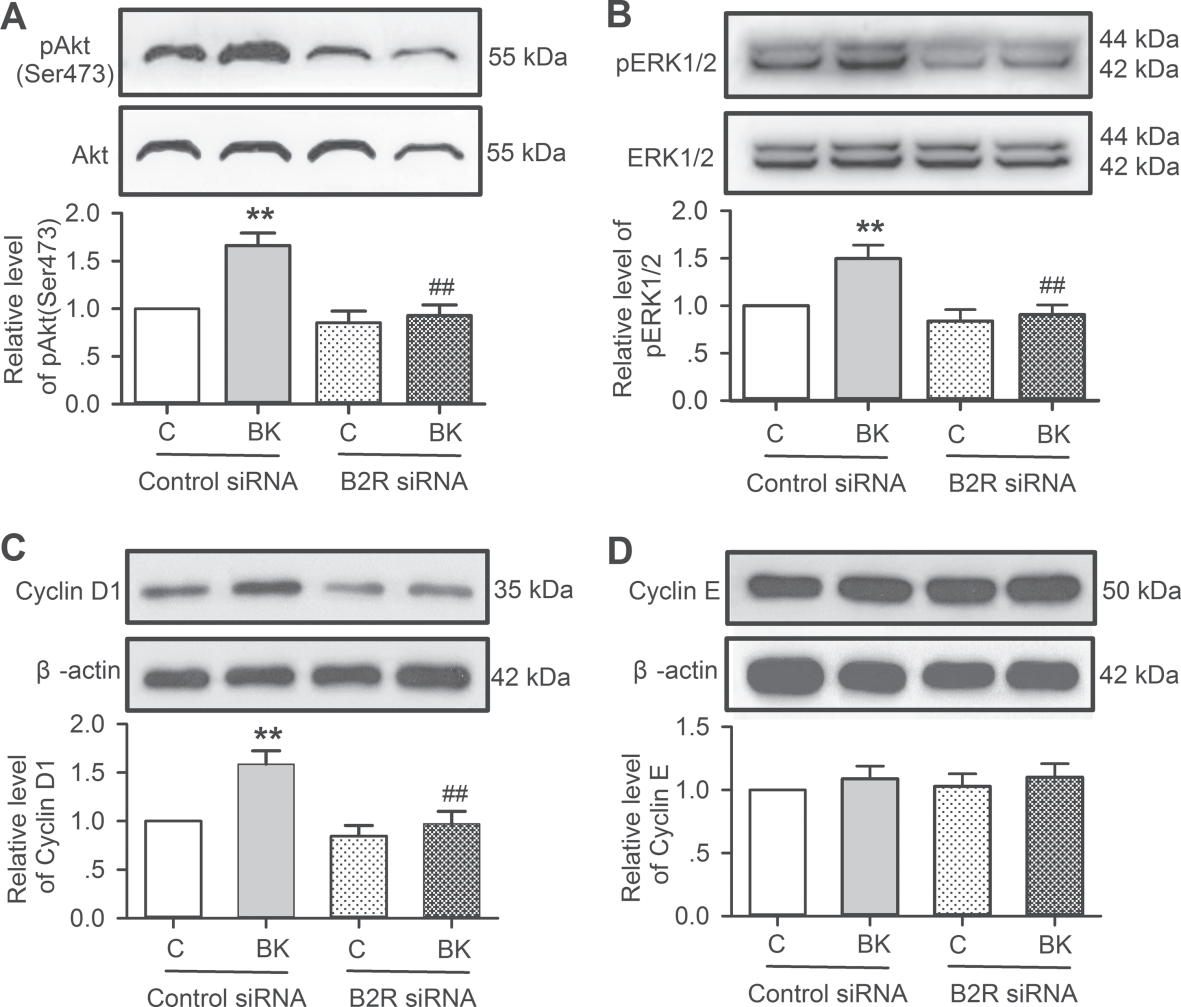
Effects of bradykinin (BK) on molecular signals in human cardiac c-Kit^+^ progenitor cells with B2Rs silenced **A**. Bradykinin (10 nM) increased pAkt level in cells transfected with control siRNA, but not with B2R siRNA. **B**. Bradykinin (10 nM) increased pERK1/2 level in cells transfected with control siRNA, but not with B2R siRNA. **C**. Bradykinin (10 nM) increased cyclin D1 level in cells transfected with control siRNA, but not with B2R siRNA. **D**. Bradykinin (10 nM) had no effect on cyclin E level in cells transfected with control siRNA or B2R siRNA. (*n* = 3, **P < 0.01 *vs* control siRNA treated with vehicle (v), ## *P <* 0.01 *vs*. control siRNA treated with 10 nM bradykinin).

The signal molecular involvement of bradykinin in increasing cell proliferation and migration was further determined in cells transfected with B2R siRNA molecules. Figure 8 shows that bradykinin-induced increase of pAkt, pERK1/2 and cyclin D1 was seen in cells transfected with control siRNA (*n* = 3, *P <* 0.01 *vs*. control), but not in cells transfected with B2R siRNA. These results confirm that bradykinin simulation of cell proliferation and migration is mediated by B2Rs followed by activation of pAkt, pERK1/2 and cyclin D1.

**Figure 9:**
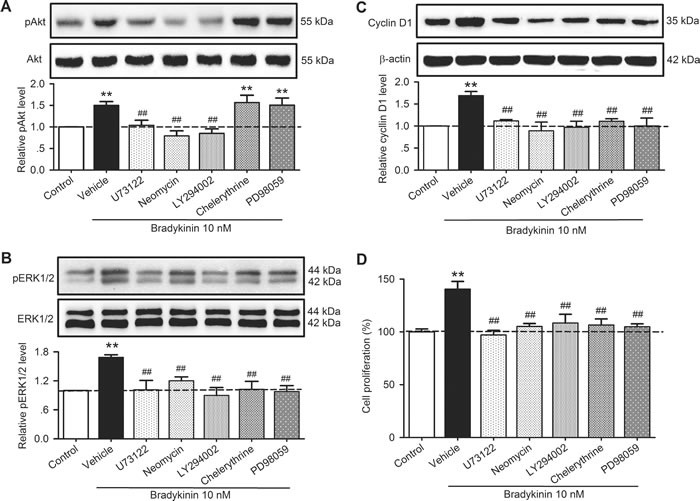
Effects of related signal antagonists on pAkt, pERK1/2 and cyclin D1 in human cardiac c-Kit^+^ progenitor cells **A**. Changes in pAkt (Ser473) in cells treated with 10 nM bradykinin in the absence (vehicle) and presence of 5 µM U73122 and 50 µM neomycin, 10 µM LY294002, 2 µM chelerythrine, and 10 µM PD98059. **B**. Changes in pERK1/2 in cells treated with 10 nM bradykinin in the absence or presence of U73122, neomycin, LY294002, chelerythrine, or PD98059. **C**. Changes in cyclin D1 protein expression in cells treated with 10 nM bradykinin in the absence or presence of U73122, neomycin, LY294002, chelerythrine, or PD98059. **D**. Increase of cell proliferation by bradykinin was fully countered by U73122, neomycin, LY294002, chelerythrine, or PD98059. (*n* = 3, **P < 0.01 *vs*. control, ##*P <* 0.01 *vs*. vehicle plus 10 nM bradykinin).

It has been reported that PLC, PI3K, PKC, and MAPK kinase mediate the activation of pAkt, pERK1/2 and/or cyclin D [18–21]. To investigate whether this is the case in human cardiac c-Kit^+^ progenitor cells, we determined the effects of bradykinin on pAkt, pERK1/2 and cyclin D in cells treated with the PLC inhibitors U73122 (5 µM) and neomycin (50 µM), the PI3K antagonist LY294002 (10 µM), the PKC inhibitor chelerythrine (2 µM), and the MAPK kinase inhibitor PD98059 (10 µM). Figure 9 illustrates the effects of these inhibitors/antagonists on pAkt, pERK1/2 or cyclin D1 in human cardiac c-Kit^+^ progenitor cells treated with 10 nM bradykinin. The increase of pAkt by bradykinin was reduced by U73122, neomycin and LY294002, but not chelerythrine or PD98059 (Figure 9A), while bradykinin-induced increase of pERK1/2 (Figure 9B) and cyclin D1 (Figure 9C) was decreased by U73122, neomycin, LY294002, chelerythrine or PD98059. These results suggest that PLC and PI3K are involved in bradykinin-induced increase of pAkt, and PLC, PI3K, PKC, and MAPK kinase are involved in the increase of pERK1/2 and cyclin D1 by bradykinin.

The effect of signaling inhibitors on cell proliferation was confirmed in human cardiac c-Kit^+^ progenitor cells. Figure 9D illustrates the percent changes in cell proliferation induced by bradykinin in the absence (vehicle) and presence of 5 µM U73122 and 50 µM neomycin, 10 µM LY294002, 2 µM chelerythrine, and 10 µM PD98059. The bradykinin-induced proliferation was fully countered in cells treated with U73122, neomycin, LY294002, chelerythrine or PD98059. These results further support the notion that PLC, PI3K, PKC, and MAPK kinase mediate the proliferation regulation by bradykinin.

## DISCUSSION

It is generally recognized that bradykinin is generated by the kinin-kallikrein system. In the plasma bradykinin is produced by activating hageman factor (Factor XII), then converting prekallikrein into kallikrein to cleave high molecular weight kininogen to release the kinin. It is also produced locally within tissues during insults such as ischemia-reperfusion injury and/or acute inflammation where kininogen is cleaved by cellular proteases (chymase, tryptase, etc.) of mast cells and basophils to liberate bradykinin [22, 23]. Bradykinin is rapidly degraded by endopeptidases, mainly angiotensin converting enzymes carboxypeptidase N and neutral endopeptidase, inside the tissues [24]. Bradykinin exerts its physiological effects *via* two types of G-protein coupled receptors known as B1Rs and B2Rs [25]. B2Rs are widely expressed in the heart, brain and spinal cord tissues while the inducible B1Rs are mainly expressed in these organs/tissues under pathophysiological conditions [26, 27]. In the present study, we demonstrated that B2Rs, but not B1Rs (which are expressed in IL-1β-stimulated human umbilical vein endothelial cells), are constitutively expressed in human cardiac c-Kit^+^ progenitor cells.

Human cardiac progenitor/stem cells are a potential cell source for myocardial repair/regeneration in patients with ischemic cardiomyopathy [5–8]. However, the cellular biology/physiology of these cells is not fully understood. Our previous studies reported ion channel expression and their roles in regulating cell proliferation and migration [28–30]. In this study, we demonstrated that bradykinin also mediates proliferation and migration of human cardiac c-Kit^+^ progenitor cells *via* activating B2Rs.

Bradykinin increases the heart tolerance for myocardial ischemia by activating B2Rs [31]. The increasing evidence suggests that bradykinin mediates the ischemic postconditioning and remote ischemic conditioning, and therefore exerts cardioprotection against ischemia/reperfusion injury [17, 32]. It is believed that bradykinin acts as an endogenous protective factor that reduces infarct size and improves post-ischemic contractile function [15, 33]. However, little is known about the role of bradykinin in long-term cardiac repair and myocardial remodeling after myocardial ischemia and/or ischemia/reperfusion injury. In this study, we found that bradykinin increased cell cycling progression by promoting G0/G1 boundary cells to S phase, and also stimulated cell migration in human cardiac c-Kit^+^ progenitor cells. The effects were inhibited in cells with treatment of the B2R antagonist HOE140 or with B2Rs silenced. These results indicate that bradykinin-induced increase of cell cycling progression and migration of cardiac c-Kit^+^ progenitor cells is mediated by B2Rs; however, whether this may participate in long-term myocardial repair and/or remodeling remains to be studied *in vivo* in the future.

Earlier studies demonstrated that bradykinin displays a wide range of biological activities in different tissues, including regulation of mitogenesis in several cell types [34]. The mitogenic effect of bradykinin mediated by G protein-coupled B2Rs involving MEK/MAPK is observed in epithelial breast cancer cells [35], bovine tracheal smooth muscle cells [36], and also human renal carcinoma A498 cells [37]. Bradykinin acts as a proliferative agent in MCF-7 cells by activating intracellular pathways including PKC, pAkt and pERK1/2 [38]. In this study, we demonstrated in cultured human cardiac c-Kit^+^ progenitor cells that bradykinin-induced increase of pAkt can be decreased by the PLC inhibitors U73122 and neomycin and the PI3K antagonist LY294002; the increase of pERK1/2 and cyclin D1 can be prevented by U73122, neomycin, LY294002, and the PKC inhibitor chelerythrine and the MAPK kinase inhibitor PD98059, which supports the notion that the activation of PI3K, PLC, PKC, MAPK, pAkt and/or pERK1/2 and cyclin D1 are involved in bradykinin-induced increase of cell cycling progression and mobility in human cardiac c-Kit^+^ progenitor cells.

Collectively, the present study demonstrates the novel information that bradykinin has mitogenic and mobility effects in human c-Kit^+^ progenitor cells *via* activating B2Rs. The activation of the G-protein-coupled receptors B2Rs promotes cell cycling progression and migration by activating PI3K, PLC, PKC, MAPK, followed by the elevation of pAkt and/or pERK1/2 and cyclin D1 levels. However, how bradykinin-mediated B2R activation mediates these pathways and their correlation remains to be further elucidated in the future.

## METERIALS AND METHODS

### Reagents

Alpha-MEM, EGF, bFGF, propidium iodide (PI), and RNAase A were purchased from Invitrogen (Hong Kong, China). HOE140, 5-Bromo-2′-deoxyuridine (BrdU), and mouse monoclonal anti-BrdU antibody were from Sigma-Aldrich (St. Louis, MO USA). Rabbit polyclonal anti-Akt, anti-pAkt(Ser473), anti-ERK1/2 and anti-pERK1/2 antibodies were from Cell Signaling (Danvers, MA, USA). Mouse monoclonal anti-BDKRB2 antibody was from Millipore (Merck Millipore, Germany). B2R siRNA, mouse monoclonal anti-cyclin D1, mouse monoclonal anti-cyclin E, HRP-conjugated goat anti-rabbit or rabbit anti-mouse IgG antibody were products of Santa-Cruz Biotechnology Inc. (Santa Cruz, CA, USA). Transwell permeable support polycarbonate membrane and other cell culture flasks and plates were purchased from Corning Inc. (NY, USA).

### Cell culture

Human cardiac c-Kit^+^ progenitor cells were isolated from human atrial specimens obtained from patients undergoing coronary artery bypass surgery. The tissue collection was approved by the Ethics Committee of Hong Kong University with informed consent as described previously [28–30, 39]. The human cardiac c-Kit^+^ progenitor cells (passages 3-8) were cultured at 37°C in 5% CO_2_ in Alpha-modified Eagle's medium (α-MEM) containing 10% fetal bovine serum and 100 U/mL penicillin and 100 μg/mL streptomycin, and supplemented with 2 mM glutamine, 5 ng/ml epidermal growth factor (EGF), and 5 ng/mL basic fibroblast growth factor (bFGF). When cells grew to 80% - 90% confluence, cells were harvested and plated onto 24-well plates for transwell assays, 96-well plates for MTT assays, and 6-well plates for wound healing assays, western blots, bromodeoxyuridine (BrdU) and flow cytometry.

### Reverse transcription polymerase chain reaction

The total RNA was extracted with TRIzol isolation system as described previously [39]. Reverse transcription (RT) was performed with the RT system (Takara Biotech, Dalian China) in a 20-μL reaction system. After the RT procedure, the cDNA was used for polymerase chain reaction (PCR) using specific primers. Primer sequences of human genes were: GAPDH primers, forward 5’-AACAGCGACACCCACTCCTC-3’ and reverse 5’-GAGGGGAGATTCAGTGTGGT-3’, BDKRB1 primers, forward 5’-TGCCAACATTTATCATCTCC-3’ and reverse 5’-AAGCCCAAGACAAACACC-3’, and BDKRB2 primers, forward 5’-CCTCACTCACATCCCACTC-3’ and reverse 5’-CACGAACAGCACCCAGA-3’. The cDNA (2 µL) was amplified by a DNA thermal cycler (Mycycler, Bio-Rad Laboratories, Hercules, CA) in a 25-μL reaction mixture containing recommended concentrations of PCR components. The amplification was performed under the following conditions: the mixture was initially denatured at 94°C for 2 min, then amplified by 30-35 repeating cycles (denaturation at 94°C for 45 s, annealing at 60 °C for 45 s, extension at 72 °C for 1 min), followed by a final extension at 72 °C for 5 min. The PCR products were electrophoresed through a 1.5% agarose gel, and the amplified cDNA bands were visualized by ethidium bromide staining. The bands images were captured by Chemi-Genius Bio Imaging System (Cambridge, UK).

### Cell proliferation assays

Cell proliferation was determined using 3-(4, 5-dimethyl-thiazol-2-yl)-2, 5-diphenyl tetrazolium bromide (MTT) and 5-bromo-2’-deoxyuridine (BrdU) incorporation assay as described previously [40, 41]. For MTT assays, cells were plated into 96-well plates at a density of ~3000 cells/well in 200 μL normal culture medium for 12 h. Different concentrations of bradykinin with or without HOE140 in α-MEM with 1% FBS were added into each well. After incubation for 48 h, 10 µL PBS buffered MTT (5 mg/mL) stock solution was applied to each well. Then the plates were incubated for additional 4 h. The medium was discarded, and 100 µL dimethyl sulfoxide (DMSO, Sigma) was added to each well. After the formazan crystals were dissolved, the optical density (OD) values (wave-lengths: test 570 nm; reference, 630 nm) of the samples were read on a μQuant microplate spectrophotometer (Bio-Tek Instruments, Winooski, VT). The percentage of OD values relative to vehicle control was used to indicate the changes in cell proliferation as shown in Figure 1D, Figure 5A and Figure 9D. For siRNA transfection, cells were transfected with corresponding siRNA molecules using Lipofectamine 2000 for 48-60 h before 10 nM bradykinin was applied for incubation.

For BrdU incorporation assay, approximately 5×l0 ^4^ cells were seeded to a 6-well plate containing a cover glass. After a 12 h culture, the cells were incubated in a medium containing 1% FBS and different concentrations of bradykinin, and then 30 µg/mL BrdU was added for 24 h. Afterward, the cells were washed for 3 times in PBS and fixed with 4% paraformaldehyde for 15 min at room temperature. Cells were washed for 3 times with PBS buffer and incubated in 2 M HCl for 15 min. After 3 additional washes with PBS buffer, 0.01% Triton X-100 in PBS was used. After blocking with 2% BSA for 1 h, cells were incubated with a primary monoclonal anti-BrdU antibody (1:200) overnight at 4°C, and then washed and incubated with a secondary FITC-conjugated antibody (1:800) under dark conditions for 60 min at room temperature. Propidium iodide (PI) (5 μg/mL) with RNAase was used for staining the cell nucleus for 15 min. Finally, images of the cells were taken with a confocal microscope (Leica TCS SP5II, Germany) and BrdU positive cells were counted for statistical analysis.

### Flow cytometry and cell cycle analysis

Cell cycle distribution of human cardiac c-Kit^+^ progenitor cells was determined using a flow cytometer (FC500, Beckman Coulter, Brea, CA). Briefly, the cells were detached using 0.25% trypsin, washed with PBS, and fixed with ice-cold ethanol (70%) overnight at 4°C. Ethanol was removed by centrifugation, and cell pellets were washed with PBS. Cells were incubated in a propidium iodide/PBS staining buffer (20 μg/mL propidium iodide, 10 μg/mL RNaseA, and 0.1% Triton X-100) at 37°C for 30 min. Data were collected using Cell Quest software (BD Biosciences, San Jose, CA, USA), and the percentages of G0/G1, S, and G2/M phase cells were calculated with MODFIT LT software (BD Biosciences).

### Cell migration

The cell mobility was determined using a wound-healing assay and a transwell assay [29]. Briefly, human cardiac c-Kit^+^ progenitor cells with or without transfecting corresponding siRNA were cultured to a confluent monolayer, and then scraped with a 200 μL pipette tip to produce acellular area for wound-healing observation. The cells were immediately washed with PBS, and then incubated with a culture medium containing 1% FBS and different concentrations of bradykinin or bradykinin plus HOE140 for 8 h. The defined areas of the wound gap were photographed under a phase contrast microscope (Olympus, Tokyo, Japan). The migrated cells on the images were counted to assess cell mobility under different treatments.

Transwell assay was performed using a modified Boyden chamber with 8 μm pore polycarbonate membranes to examine the potential effect of bradykinin on cell migration. The chambers were pre-coated with 600 µL serum-free medium for at least 1 h. After removing pre-coating medium, around 5000 cells with or without siRNA transfection were plated into the upper chamber with 200 µL medium containing 1% FBS, and 600 µL medium with 1% FBS and different concentrations of bradykinin was maintained in the lower chamber. The plates were incubated at 37°C in 5% CO_2_ for 8 h. Then the chambers were washed three times with PBS, fixed with formaldehyde for 15 min at room temperature, and stained with crystal violet for 15 min. After washing thoroughly with PBS to remove the dye, non-migrated cells on the upper surface of the membrane were scraped off by cotton swabs. The migrated cells on the lower surface of the membrane were counted in 5-8 representative fields under a microscope.

### Western blot analysis

Western blot was performed as previously described [42]. Briefly, cells were lysed with a modified RIPA buffer with various protease inhibitors. Protein concentration was determined by Bio-Rad protein assay. Cell lysates were mixed with sample buffer and denatured by heating to 95°C for 5 min. Samples were resolved *via* SDS-PAGE and transferred to nitrocellulose membranes. Membranes were blocked with 5% non-fat milk in Tris Buffer Saline with 0.1% Tween-20 (TBST) then probed with primary antibodies (1:1000-2000) at 4°C overnight with agitation. After washing with TBST, the membranes were incubated with HRP-conjugated goat anti-rabbit or rabbit anti-mouse secondary antibody at 1:5000 dilutions in TBST at room temperature for 1 hr. Membranes were washed again with TBST then processed onto x-ray films using an enhanced chemiluminescence detection system. The relative band intensities were measured by quantitative scanning densitometer and image analysis software.

### RNA interference

Small-interfering RNA (siRNA) molecules targeting human B2R mRNA was purchased from Santa Cruz Biotechnology. This siRNA is target-specific 19- to 25-nt siRNA designed to knock down mRNA expression as previously described [29, 39]. Human cardiac c-Kit^+^ cells at 60 - 80% confluence in 96-well plates or 6-well plates were transfected with siRNA molecules at 10-40 nM using Lipofectamine 2000 reagent (Invitrogen). The silencer negative control siRNA, which has no known target in human genomes, was applied as negative control. 48 to 72 h after transfection, cells were treated with/without 10 nM bradykinin then used for determining B2R mRNA and protein expression, cell proliferation, cell cycle assay, as well as migration assays or western blot for determining signaling pathway.

### Statistical analysis

Results are presented as mean ± S.E.M. Unpaired Student's *t*-tests were used as appropriate to evaluate the statistical significance of differences between two group means, and one-way ANOVA followed by the Tukey-test was used for multiple comparisons. Values of *P* < 0.05 were considered statistically significance.
